# Combination of percutaneous left appendage epicardial ligation and endo-epicardial atrial fibrillation ablation

**DOI:** 10.3389/fcvm.2023.1224924

**Published:** 2023-10-09

**Authors:** Stefano Grossi, Francesca Bianchi, Alessandro Blandino, Chiara Pintor, Antonino Tomasello, Barbara Mabritto, Giuseppe Musumeci

**Affiliations:** ^1^ Cardiology Department, AO Ordine Mauriziano, Turin, Italy; ^2^ Biosense Webster Italy, Johnson&Johnson MedTech Italy, Pomezia (RM), Italy

**Keywords:** atrial fibrillation ablation, epicardial ablation, left appendage occlusion, left appendage ligation, transcatheter ablation

## Abstract

**Introduction:**

Atrial fibrillation (AF) is the main cause of cardioembolic stroke. In high-bleeding-risk patients, long-life anticoagulation therapy is not permitted, and left atrial appendage (LAA) closure may be considered. LAA is also a critical substrate for AF. Epicardial LAA occlusion has several advantages: LAA ligation results in a favorable electrical and structural atrial remodeling, which decreases AF recurrence. Endocardial ablation alone is not efficient for all patients, and new evidence shows better outcomes in patients affected by persistent AF after a combined hybrid endo-epicardial ablation. Considering the synergic potential of these techniques, in this case series, they were both combined in a single procedure.

**Methods and results:**

We describe the treatment of 5 patients referred for refractory AF ablation and LAA closure. All patients had high thrombotic and previous major hemorrhage, with relative contraindication to life-long therapy with anticoagulation. A combined procedure of LAA ligation and endo-epicardial ablation was scheduled with short-term anticoagulation. LAA closure was performed with an epicardial approach using the LARIAT system. Then, LA mapping and ablation were performed, endocardially and then epicardially.

All procedures were concluded without complications.

At follow-up, in all patients, transesophageal echocardiography showed the complete occlusion of the LAA; therefore, anticoagulation therapy was interrupted. All patients were asymptomatic, and in the sinus rhythm, no hemorrhage or ischemic events occurred.

**Conclusion:**

The combination of percutaneous LAA ligation and endo-epicardial ablation was revealed to be feasible and safe and might represent a new approach for the treatment of refractory AF in patients with indication of LAA occlusion.

## 1. Introduction

Atrial fibrillation (AF) is one of the main causes of cardioembolic stroke. After the introduction of direct oral anticoagulants (DOACs), treatment adherence increases; however, in high-bleeding-risk patients, long-life anticoagulation therapy is still not permitted, and left atrial appendage (LAA) closure may be considered (1, 2).

Moreover, LAA is a critical substrate for AF maintenance by itself, sometimes involved in the failure of traditional percutaneous ablation procedures (3).

Epicardial left atrial appendage occlusion has several advantages over endocardial devices: LAA ligation (4–6) results in favorable electrical and structural atrial remodeling, which decreases atrial fibrillation recurrence.

It has also been demonstrated that the endocardial ablation approach alone is not effective in all patients (7), and new scientific evidence, though limited, shows better outcomes in patients affected by persistent atrial fibrillation after a combined endo-epicardial approach (8–10).

A single-stage endo-epicardial AF ablation (9, 10) results in favorable rhythm and symptom outcomes, allowing a greater transmural understanding of the AF substrate.

Considering the synergic potential of these techniques, in this case series, they were both combined in a single fully percutaneous, non-surgical procedure.

## 2. Methods

### 2.1. Patient population

In this case series, we describe the treatment of five patients referred for symptomatic refractory AF to at least one antiarrhythmic drug and at least one previous transcatheter ablation procedure and contra-indication to life-long anticoagulation therapy and high thrombotic risk (CHA2DS2-VASc score > 2). We describe in this series only patients who had completed a follow-up of at least 12 weeks (see Table 1).

**Table 1:** The table describes the patient population considered in the case series (PVI: pulmonary veins isolation).

Patient number	Gender	Number of previous procedures	Previous ablation scheme	CHADSVASC	HASBLD	Follow-up (months)
1	M	2	PVI Roof line	3	3	14
2	M	2	PVI Roof line Posterior wall	3	3	10
3	F	2	PVI Right isthmus	5	4	9
4	M	3	PVI Right isthmus Roof line	3	3	8
5	M	1	PVI	3	3	6

A combined procedure of LAA ligation and endo-epicardial ablation was scheduled with a short-term anticoagulation protocol of 4 weeks before and 8 weeks after the procedure.

All patients underwent a pre-operative gated computed tomographic (CT) angiography for determination and three-dimensional (3D) reconstruction of cardiac and thoracic structures demonstrating an LAA anatomy and position suitable with the LARIAT device (SentreHeart, CA, USA) (6, 11, 12).

### 2.2. Procedure description

After informed consent, a stepwise procedure was performed: first, epicardial LAA closure with the LARIAT system under fluoroscopic and transesophageal echocardiographic guidance (as described below in paragraph 2.2.1 and in previous studies (4–6); then, epi-endocardial ablation was completed.

Transesophageal echocardiography was performed before the procedure to evaluate the left atrium and LAA and rule out the presence of thrombosis and then used for cardiac monitoring during the procedure to guide and assess LAA closure.

An electro-anatomical endocardial map of the right atrium and right ventricle was reconstructed with the 3D mapping system CARTO® 3 (Biosense Webster, Diamond Bar, CA) and synchronized with a pre-acquired CT scan (CARTOSeg^™^) and fluoroscopic image (CARTOUnivu^™^).

#### 2.2.1. LAA closure

Epicardial puncture was achieved using a telescopic needle. Two guides were positioned in the pericardium, the subxiphoid access was progressively dilated, and a dedicated pericardial introducer was positioned.

After epicardial access was safely obtained, without bleeding, a transeptal puncture was performed, and the LAA morphology was visualized by angiography. An electro-anatomical endocardial map of the left atrium (LA) was reconstructed with the CARTO® 3 system, and synchronization with the pre-acquired CT scan was further verified.

Then, the dedicated magnetic tip guides were advanced: the first one in the epicardial space anterior to the LAA and the second one via the transeptal approach. The magnets were coupled, and the correct positioning was verified.

**Figure 1:**
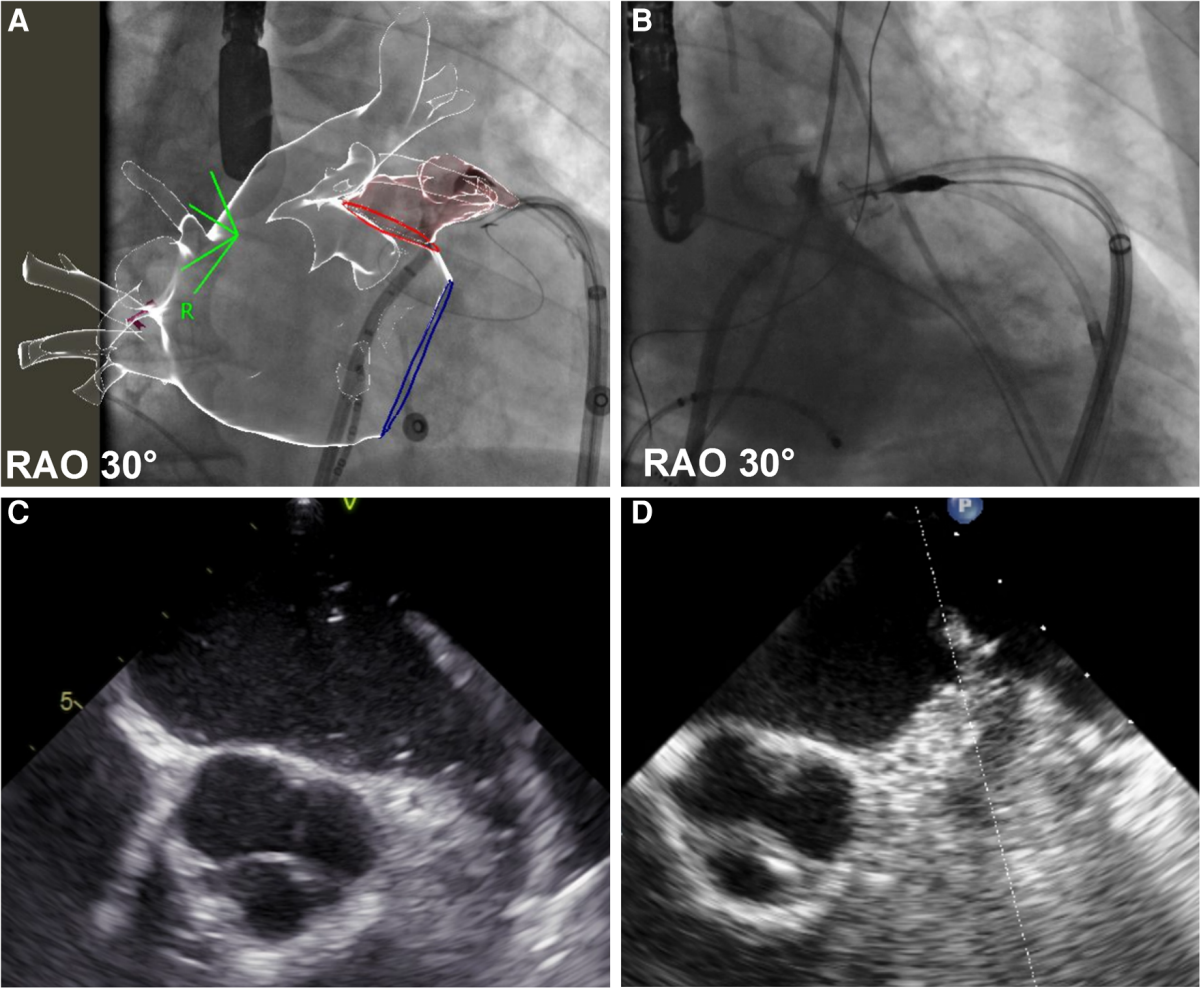
Before and after left appendage ligation. The figure shows left atrial appendage (LAA) angiography before (panel **A**—fluoroscopic image, panel **C** echocardiographic frame) and after (panel **B**—fluoroscopic image, panel **D** echocardiographic frame) LAA ligation. In panel **A**, fluoroscopic images are synchronized with the CT scan reconstruction, previously merged with the electro-anatomical map of the left atrium. Detailed description in text.

The LARIAT “noose device” was advanced through the epicardial introducer up to the LAA ostium, and angiography was repeated. Then, the loop was closed with echocardiographic and angiographic demonstration of the complete LAA occlusion (see Figure 1).

Finally, the suture was carried out using a dedicated device, the epicardial system was retracted, and the pigtail drain was left in pericardium.

#### 2.2.2. Mapping and ablation

LA mapping was performed endocardially and then epicardially: a substrate bipolar map during AF or sinus rhythm was formed using a 3D mapping system with image integration, both with a fluoroscopic image and pre-acquired CT.

If pulmonary veins (PV) reconnection was detected, antral circumferential PV ablation was completed using a contact-force, irrigated radiofrequency (RF) catheter (ThermoCool SmartTouch^™^, Biosense Webster Inc.) guided by the CARTO VISITAG^™^ Module with Ablation Index. The software provides a built-in algorithm and a standard workflow, showing a single value (index) during ablation, which represents the integration of the catheter stability during RF application, RF power (40 W posteriorly, 45 W anteriorly), contact force (5–20 g), and time. The software enhances the creation of reproducible lesions by reaching a specific index target.

An esophageal probe was displayed on the angiographic system integrated with the mapping system. The epicardial map was then created: if electrical activity was still present on the epicardial aspect of the antral endocardial isolated area, radiofrequency application (power-controlled mode at 40 W with ThermoCool SmartTouch^™^, Biosense Webster Inc.) was delivered from the epicardial side to complete PV isolation (PVI).

Phrenic nerve capture was excluded with pacing maneuvers, and adequate (>2 cm) distance from the coronary arteries and esophagus was verified at any site before epicardial RF delivery.

Linear ablation was then endocardially performed: the roof line and postero-inferior line in all patients.

**Figure 2:**
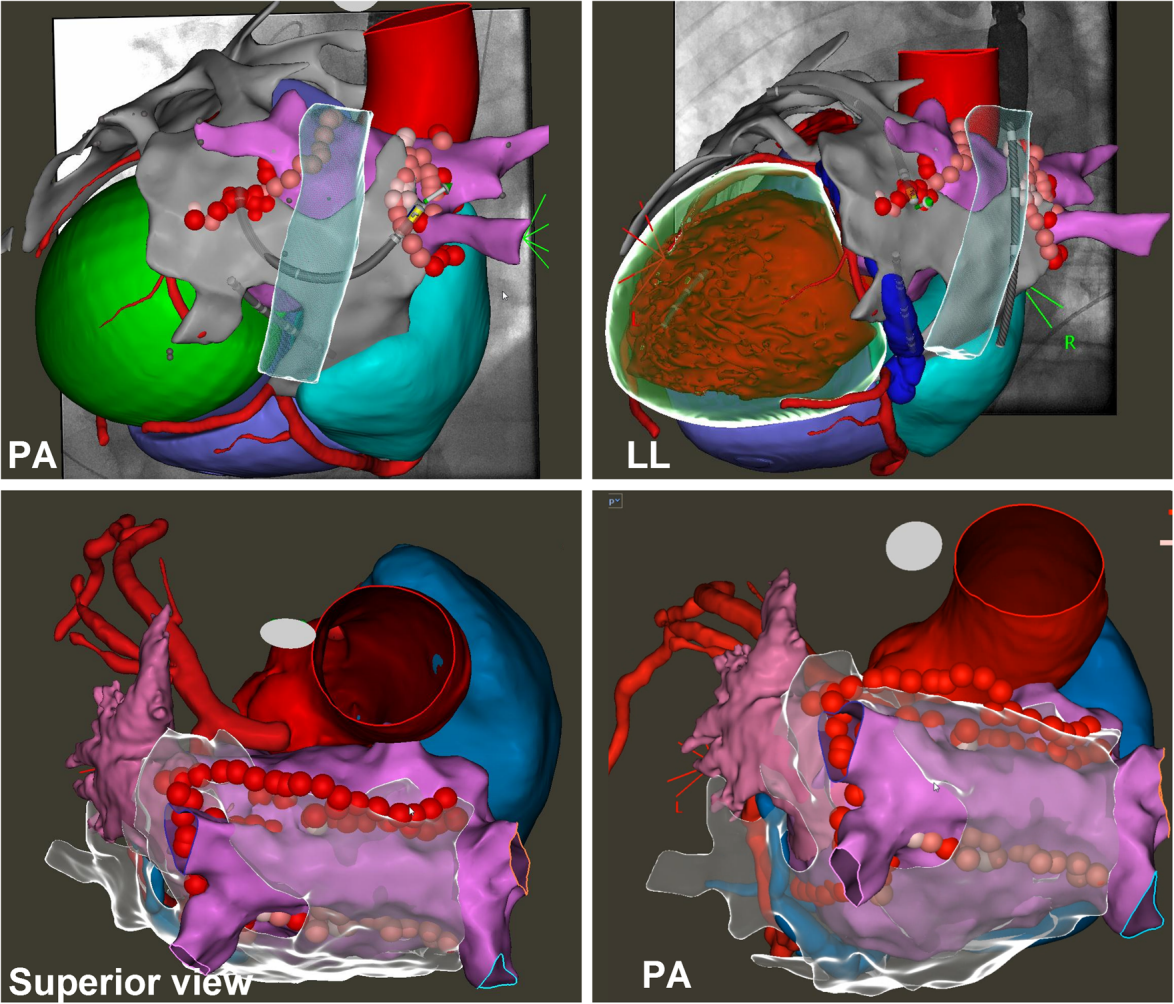
Endo-epicardial atrial fibrillation ablation. The images show the 3D electroanatomical left atrium (pink) and epicardium (grey) maps synchronized with the CT scan and fluoroscopic images. On the maps, it is possible to see the ablation lines performed both endocardially (PVI, roof line, and posterior line) and epicardially (PVI, roof line, and left isthmus line). See text.

**Figure 3:**
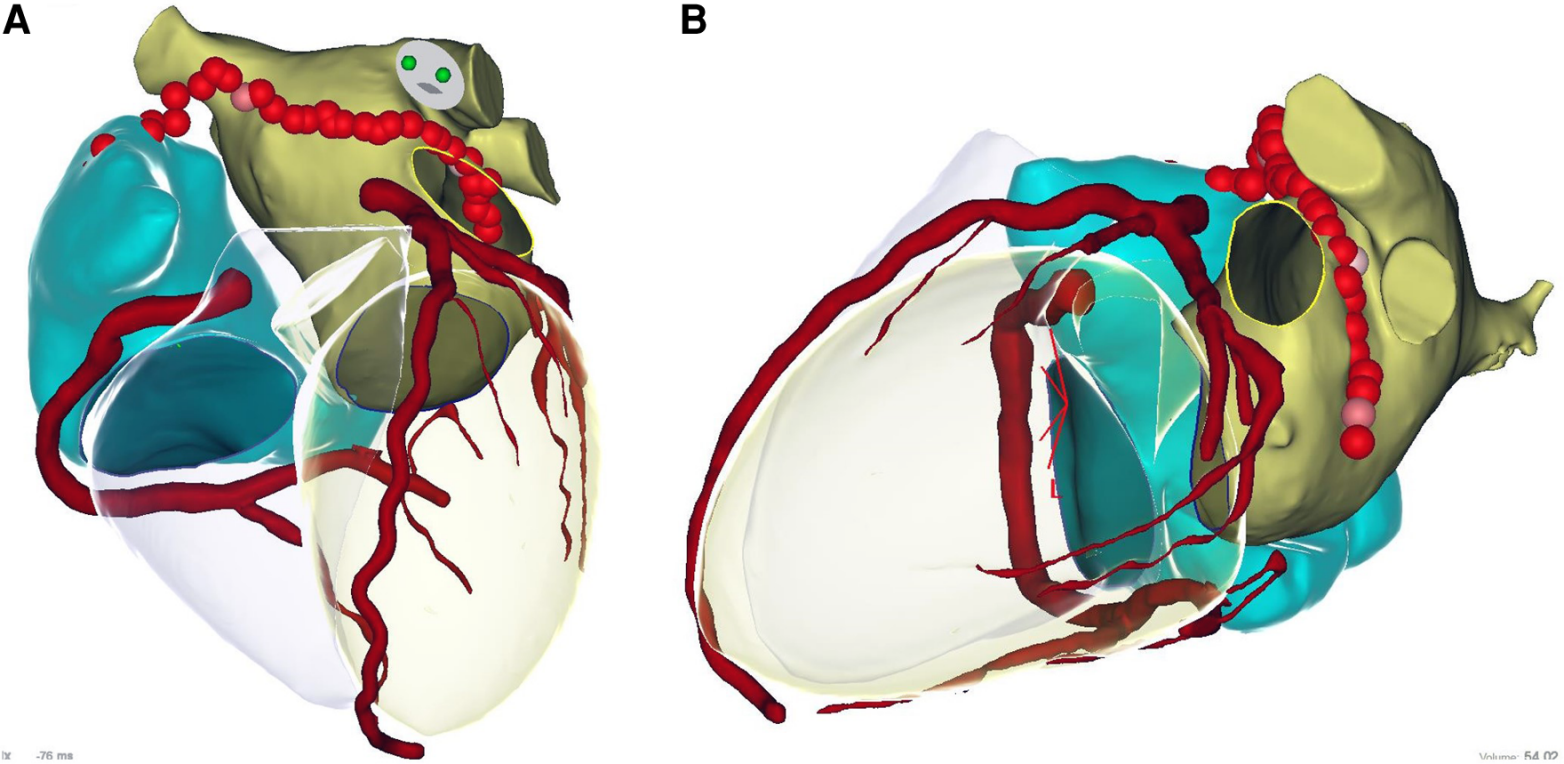
Epicardial linear lesions. Left panel (**A**): roof line from antero-posterior view merged with CT reconstruction. Right panel (**B**): lateral view merged with CT reconstruction of the ablation line performed anteriorly to the left pulmonary veins and Marshall ligament. See text. In the CT scan reconstruction, the following structures are displayed: left atrium (yellow), right atrium (light blue), coronary arteries (red), and left and right ventricle (transparent).

An epicardial ablation line was then performed starting from the superior vena cava, including the Bachman bundle, through the roof following the endocardial line and to the ridge between the appendage and left pulmonary veins: the anterior epicardial aspect of the encircling left pulmonary veins and the Marshall ligament (see Figures 2, 3 and Table 2).

**Table 2:** The table describes the ablation scheme for each patient.

Patient number	Endocardial lesions	Epicardial lesions
1	PVI Roof line Postero-inferior line	PVI Epicardial roof line^a^
2	PVI Roof line Postero-inferior line	PVI Epicardial roof line^a^
3	Roof line Postero-inferior line	PVI Epicardial roof line^a^
4	Roof line Postero-inferior line	PVI Epicardial roof line^a^
5	PVI Roof line Postero-inferior line	PVI Epicardial roof line^a^

^a^ The “epicardial roof line” is intended from the superior vena cava, including the Bachman bundle, through the roof following the endocardial line and to the ridge between the appendage and left pulmonary veins.

The posterior left atrial wall was not targeted for epicardial ablation due to the presence of the esophagus.

Finally, PVI and line blocks were demonstrated by mapping and pacing maneuvers.

At the end of the procedure, all patients received intrapericardial methylprednisolone.

### 2.3. Post-procedural management

The pericardial drain was removed when drainage was less than 50 ml over a 12-h period, and there was no effusion detected through echocardiography.

Oral anticoagulant therapy was early restarted 6 h after the end of the procedure if no effusion was detected through echocardiography and drainage was less than 30 ml, and it was maintained until the first follow-up scheduled at 8 weeks.

All patients were prescribed post-operative prophylactic colchicine (0.5 mg once a day in patients <70 kg, twice a day if >70 kg) for at least 4 weeks and prednisone with a scaled-down dose for a cumulative 4 weeks.

## 3. Results

There were five patients in total with a mean age of 64.4 ± 3 years, comprising four men and one woman, who were treated with combined percutaneous endo-epicardial ablation and appendage ligation.

They all suffered from AF refractory to at least one antiarrhythmic drug and at least one previous transcatheter ablation procedure (four had been previously submitted to ≥2 left atrial ablation for AF). All patients had high thrombotic (CHA2DS2-VASc score > 2) and high bleeding risk, with relative contraindication to life-long therapy with anticoagulation due to previous major hemorrhage (two patients suffered from subarachnoid hemorrhage in the past and three from repeated bleeding due to intestinal angiodysplasia).

Endo-epicardial mapping and ablation and appendage ligation were performed in the electrophysiology laboratory. In four patients, it was performed under deep sedation in spontaneous breathing, and in one patient suffering from known lung disease, the procedure was performed under general anesthesia with orotracheal intubation.

All procedures were successfully concluded with complete appendage exclusion after epicardial ligation (see Figure 1), as demonstrated with both transesophageal echocardiography and angiography.

The mapping and ablation scheme was performed as illustrated in the Methods and is shown in Figures 2, 3.

During the endocardial mapping, in three of five patients the pulmonary veins were found to be isolated from the previous ablation procedure; in the other two, electrical reconnection was recorded, and therefore, wide circumferential ablation was performed.

In all patients during epicardial mapping on the site of PVI, more or less extensive areas of epicardial breakthrough were found, and therefore, electrical isolation of the veins was completed on the epicardial side: particularly in the anterior region of the ostium of the left pulmonary veins, electrical activity was always recorded on the epicardial front even after ligation of the appendage.

Particularly, breakthroughs were identified by combining endo-epicardial bipolar maps and pacing maneuvers in the sinus rhythm: from the endocardial side, pacing was performed at 5 V, and no capture was detected, whereas pacing at a 10 V capture was obtained, but no endocardial potentials were visible.

At the level of the roof line of the atrium, even after linear endocardial ablation electrical activity on the epicardial side was still recorded, the roof line was targeted to linear ablation according to the above-described scheme.

One patient with long-lasting persistent AF was converted with external DC shock after completion of the ablation scheme. One patient was in sinus rhythm from the beginning of the procedure; in three patients, during epicardial linear ablation, AF organized to atypical atrial flutter that was subsequently ablated at the level of Marshal ligament in two patients and of Bachman bundle in the other one.

Bidirectional conduction block across all linear lesions and pulmonary veins circumferential entrance and exit blocks were verified after sinus rhythm restoration by pacing in all patients.

No periprocedural or late complications occurred in all patients.

The mean procedural time was 180 min ± 30.

Pericardial drainage was safely removed after 12 h of monitoring and echocardiographic check.

All patients were discharged on the third day without complications. All were prescribed prophylactic anti-inflammatory therapy with colchicine and prednisone, as described above.

At the first follow-up at 8 weeks, in all patients, transesophageal echocardiography confirmed the complete occlusion of the left atrial appendage, with no leakage or doppler flow signals, and therefore, anticoagulation therapy was interrupted.

At the subsequent follow-up of 6 ± 2 months, all patients were asymptomatic, and in the sinus rhythm, neither hemorrhage nor ischemic events were registered.

## 4. Discussion

Catheter ablation is a cornerstone in the therapy of AF. Nonetheless, the success rate of endocardial ablation is far from optimal, particularly in patients with persistent AF and in the setting of structural heath disease.

Failure occurrence can be explained by the presence of gaps along the ablation lesion, both in a planar and a transmural dimension.

Surgical and hybrid approaches have been systematically shown to achieve better results than endocardial catheter ablation (12, 13), including epicardial ablation and LAA ligation.

LAA exclusion is crucial in stroke prevention in patients with contraindication to anticoagulation therapy, but it is also effective in AF recurrence prevention due to the extrapulmonary foci abolition and critical mass reduction. In a previous series of 33 patients highly refractory to previous endocardial ablation, the patients submitted to a hybrid procedure with a surgical subxiphoid approach and epicardial ablation of AF and underwent LAA ligation with Lariat System together with endocardial PV isolation. The success rate of the sinus rhythm maintenance was above 90% in the follow-up (10).

In recent years, the increased familiarity with the percutaneous epicardial approach, gained by the mapping and ablation of ventricular arrhythmias, has enabled electrophysiologists to achieve endo-epicardial ablation of atrial fibrillation, which has proven to be a safe and effective method for selected patients with refractory AF (9, 14).

Our aim was to perform endo-epicardial ablation combined with LAA ligation in a purely percutaneous procedure in patients with AF refractory to both previous endocardial ablation and pharmacological therapy.

The feasibility and safety were demonstrated by the accomplishment of the procedure in all cases without complication. The effectiveness of the procedure was assessed by the absence of arrhythmic recurrences in the subsequent follow-up.

After endocardial PVs isolation, epicardial mapping highlighted the persistence of epicardial connections through the encircling lines. Transmural gaps were also evident along the roof line.

Radiofrequency ablation on the epicardial aspect of the left atrial wall enabled to abolish conduction along the lines, as confirmed by the pacing maneuvers. Our ablation scheme also involved the Bachman bundle and the sulcus between the left superior pulmonary vein and Marshall ligament: all these structures are responsible for recurrent post-ablation left atrial or bi-atrial flutter often restistent to endocardial ablation. The endo-epicardial approach enabled a conduction block along these thick fibers.

Besides the effects on electrical conduction, the cardiac autonomic nervous system got involved by epicardial radiofrequency delivery, and this may contribute to the ablation success, although its mechanisms remain to be completely clarified.

In our patient series, a contraindication to long-term oral anticoagulation was present despite the elevated cardioembolic risk. The LARIAT device ligation obtained complete LAA exclusion in the acute phase in all cases, which persisted at follow-up, enabling anticoagulation to be stopped.

Moreover, although traditionally the role of LAA in arrhythmogenesis was poorly understood, LAA has come to the forefront, with several studies showing the role of LAA in atrial arrhythmogenesis.

In a previous study, it was assessed that mechanical LAA exclusion with the LARIAT device appears to be more efficacious than one-time endocardial LAA radiofrequency electrical isolation (9).

We, therefore, believe that in our study, the LARIAT LAA ligation also provided an antiarrhythmic effect, both eliminating a trigger source and reducing atrial critical mass.

## 5. Limitations

Although the left posterior atrial wall often shows a non-uniform epicardial activation after a box isolation attempt from the endocardium, in our approach, no additional epicardial ablation was added taking into account a safety issue due to esophagus proximity.

Thus, septo-pulmonary epicardial fibers were not included in the ablation scheme and could contribute to arrhythmic recurrences.

The follow-up data are relatively short, and the exclusion of recurrences was assessed with 24 h Holter monitoring.

## 6. Conclusion

This pilot study, performed in selected patients, shows that the combination of purely percutaneous left appendage epicardial ligation and endo-epicardial ablation of AF is feasible and safe and might represent a new approach for the treatment of refractory atrial fibrillation in patients with high hemorrhagic and thromboembolic risk.

Due to the possible role played by LAA in AF maintenance, it can be hypothesized that combining LAA ligation with endo-epicardial AF ablation could be, in the future, also extended to patients with refractory persistent AF and/or dilated atria, even in the absence of conventional LAA closure indication.

## Data Availability

The raw data supporting the conclusions of this article will be made available by the authors, without undue reservation.
